# Absorption and Distribution of Imidacloprid and Its Metabolites in Goldfish (*Carassius auratus* Linnaeus)

**DOI:** 10.3390/toxics11070619

**Published:** 2023-07-17

**Authors:** Wanghui Xu, Lulu Zhang, Jiayin Hou, Xiaohua Du, Liezhong Chen

**Affiliations:** 1 College of Chemical Engineering, Zhejiang University of Technology, Hangzhou 310014, China; 2 State Key Laboratory for Managing Biotic and Chemical Threats to the Quality and Safety of Agro-Products, Zhejiang Academy of Agricultural Sciences, Hangzhou 310021, Chinajiayinhou@163.com (J.H.); 3 College of Food and Pharm Aceutical Science, Ningbo University, Ningbo 315800, China

**Keywords:** imidacloprid, goldfish, metabolites, tissue distribution, toxicity

## Abstract

Imidacloprid (IMI) is the first-generation neonicotinoid insecticide. But, the long-term use of IMI as a pesticide has caused severe water pollution. Recently, the toxicity of IMI to aquatic organisms has received increasing attention. This study aimed to investigate the absorption and distribution of IMI in various tissues (gills, intestine, liver, muscle, brain and gonads) of goldfish through short-term and continuous exposure tests over 28 days. The results of short-term exposure indicated that the concentration of IMI and its metabolites in tissues at the transfer stage decreased steadily after 1 day of 40 mg/L IMI water treatment and was below the detection limit after 3 days. Continuous exposure for 28 days at various treatment concentrations showed that the concentrations of IMI and its metabolites differed significantly among the different tissues of the goldfish. In the 20 mg/L treatment group (S1), the highest concentration of IMI was found in the liver (12.04 μg/g_tissue_), followed by the intestine (9.91 μg/g_tissue_), muscle (6.20 μg/g_tissue_), gill (6.11 μg/g_tissue_), gonads (5.22 μg/g_tissue_) and brain (2.87 μg/g_tissue_). In the 40 mg/L treatment group (S2), the order of the tissue concentrations was similar to that of the S1 group, with the highest concentration observed in the liver (12.04 μg/g_tissue_), followed by the intestine (9.91 μg/g_tissue_), muscle (6.20 μg/g_tissue_), gill (6.11 μg/g_tissue_), gonads (5.22 μg/g_tissue_) and brain (2.87 μg/g_tissue_). Furthermore, the study detected 5-hydroxy-IMI, IMI urea and 6-chloronicotinic acid in IMI metabolites in all tissues, while IMI was detected only in the intestine and liver. Overall, the results of this study contribute to a better understanding of the metabolic behavior of IMI in organisms and provide new data to support the assessment of IMI toxicity in fish.

## 1. Introduction

Imidacloprid (IMI) is the first-generation neonicotinoid insecticide developed by Bayer Crop Science, Leverkusen, Germany [1]. It is mainly used for the control of aphids, leaf hoppers, thrips and other stinging mouthparts pests. The mechanism of its insecticidal action is to act on the nicotinic acetylcholine receptors in the postsynaptic membrane of the insect nervous system and its surrounding nerves so that the insects can maintain continuous excitement, paralysis and then die [2,3]. The effect of IMI is relatively fast, and it will have a strong control effect on pests 1 day after the drug. Compared with traditional pesticides, IMI has the advantages of high efficiency, high selectivity and lasting effect on target pests, and it was widely used all over the world soon after its launch.

However, in the application process of IMI, only a small amount of the effective components is absorbed by crops (approximately 5%), most of which will enter the soil and eventually enter the water environment with infiltration, runoff and other methods [4]. Because the water solubility, stability and persistence of IMI and their residues have been detected in water bodies worldwide [5], Struger et al. conducted three consecutive years of sampling and detection in the surrounding surface waters of 15 agricultural active areas in southwestern Ontario, Canada, from 2012 to 2014, and the results showed that the detection rate of IMI exceeded 90% in more than half of the areas. In 75% of samples collected in two regions, the concentration of IMI exceeded the local legal limit (230 ng/L) [6]. In California, Starner Keith et al. collected and tested 75 surface water samples from rivers, creeks and drains around farmland, and the results showed that IMI was detected in 67 samples, with a maximum residue of 3.29 μg/L and an average concentration of 0.77 μg/L [7]. Similarly, IMI residues have been detected in water bodies of various basins in China, such as the Yangtze River and the Yellow River, with detection concentrations ranging between 2.08 ng/L and 121.71 ng/L and an average detection concentration of 41.89 ng/L [8]. In conclusion, IMI has been detected in lakes, rivers, groundwater and other water bodies at home and abroad to varying degrees, and some areas even seriously exceed the standard. The contamination of IMI in aquatic environments may cause potential health hazards to aquatic organisms. Fish are an important part of aquatic life, so it is necessary to assess the potential harm of IMI to fish.

Researchers previously believed that IMI had low toxicity to nontarget organisms and lacked teratogenic, carcinogenic and mutagenic effects [9]. Whitehorn et al. discovered that the use of IMI could significantly inhibit the reproductive ability of bumblebee populations, bringing attention to the safety of IMI on nontarget organisms [10]. Subsequently, more studies have shown that IMI has certain effects on nontarget organisms. For example, low concentration of IMI can induce intestinal histological damage and intestinal oxidative stress in zebrafish, significantly increase the levels of superoxide dismutase (SOD) and catalase (CAT), and slightly induce intestinal flora imbalance and specific bacterial changes [11]. Topal et al. studied the neurotoxicity of IMI on the brain tissue of rainbow trout. The results showed that under the treatment of 10 mg/L and 20 mg/L IMI, the activity of acetylcholinesterase (AChE) decreased and the activity of 8-hydroxy-2-deoxyguanosine (8-OHdG) (a marker related to cellular oxidative stress) increased in the brain tissue of rainbow trout. Moreover, oxidative stress parameters of rainbow trout were changed, thus showing neurotoxicity to rainbow trout [12]. In addition, studies have shown that IMI can also slow down the growth rate [13], reduce activity [14], damage DNA [15] and other effects on nontarget organisms.

Goldfish (*Carassius auratus*) is a kind of carp fish and an aquatic organism with Chinese characteristics, so this study selected golden crucian carp as the experimental organism. According to the preliminary test, when the concentration of IMI is greater than 40 mg/L, golden crucian carp will die. It can be seen that 40 mg/L is the maximum tolerance concentration of golden crucian carp to IMI. The maximum tolerance concentration is the highest dose that an animal can tolerate without causing death in the animal. Therefore, two exposure concentrations (maximum tolerance concentration and 1/2 maximum tolerance concentration (40 mg/L and 20 mg/L)) were set, respectively, to study the metabolic distribution of IMI in golden crucian carp. These findings will help to understand the metabolism of IMI in fish and its effect on fish health, and they will provide new data support for the toxicity study of IMI.

## 2. Materials and Methods

### 2.1. Chemicals, Instruments and Animals

The reagents used in the experiment included IMI (purity 95.00%) from Yuanye Biotechnology Co., Ltd., Shanghai, China; IMI standard (purity 98.00%) from Anpu Experimental Technology Co., Ltd., Shanghai, China; and IMI-urea methanol standard solution (100.00 mg/L), IMI-olefin methanol standard solution (100.00 mg/L), 5-hydroxy-IMI methanol standard solution (100.00 mg/L), and 6-chloronicotinic acid methanol standard solution (100.00 mg/L) from Alta Technology Co., Ltd., Tianjin, China. Chromatographic-grade methanol and ethyl acetate were obtained from Millipore, and purified water was provided by Watson Co., Ltd., Hong Kong, China. Anhydrous magnesium sulfate (analytical grade) was sourced from Pharmaceutical Group Co., Ltd., Shanghai, China and the diatomite was from McLean Biochemical Technology Co., Ltd., Shanghai, China.

The instruments utilized in the experiment included an Albrecht sciex5500+ high-performance liquid-phase triple quadrupole tandem mass spectrometer, a Sartorius BSA124S one ten-thousandth scale from Sedolis, Göttingen, Germany, and an LC-DCY-12SF water bath nitrogen blowing instrument from Lichen Instrument Technology Co., Ltd., Shanghai, China.

The test organism for the experiment was 3-month-old goldfish purchased from Hangzhou Fengqi Flower and Bird Market. The goldfish were domesticated in the laboratory for over a week prior to the formal experiment with tap water treated by aeration dechlorination and meeting the provisions of the fishery water quality standard (GB11607-1989) used as the test water [16]. The domestication conditions were maintained at a pH of 7.0–8.5, temperature of 20.9 ± 0.4 °C, and dissolved oxygen of 6.9 ± 0.2 mg/L. In addition, a random sample of goldfish was taken according to the instrument condition 2.4 before exposure began, and it was found that IMI and its metabolites were not present in their bodies.

### 2.2. Exposure Experiment

The test was divided into two parts: short-term exposure and long-term sustained exposure.

Short-term exposure test: 60 domesticated goldfish were placed into a 20 L culture barrel with 15 L of test water containing 40 mg/L IMI. The goldfish in the control group were cultured with fully aerated and chlorinated tap water. After 1 day of exposure, the golden crucian carp were transferred to clean water for feeding. During feeding, the breeding density was maintained at 4 fish/L, and the test water was updated daily to check for abnormal behavior and mortality. They were fed twice a day, and soon after feeding, the uneaten food and feces were sucked out of the culture bucket to avoid food absorption and adsorption. At 0 h, 0.5 d, 1 d, 1.5 d and 3 d after transfer, 5 fish were randomly selected from the experimental group (each fish was an independent sample), and the liver, intestine, muscle, gill tissue, brain tissue and gonads of the golden carp were dissected in an EP tube. The collection method for the golden crucian carp tissues refers to the collection method for the cyprinid fish in Zheng’s study [17]. After collection, the tissue weight was recorded, ground into a chymous shape with a tissue grinder, and stored at −80 °C until analysis.

The exposure experiment lasted for 28 days. The control group was treated with fully aerated dechlorinated tap water, and the exposure group was treated with aerated dechlorinated tap water containing IMI; the exposure concentration of IMI was 20 mg/L and 40 mg/L. The feeding conditions were the same as in the short-term exposure tests. The golden crucian carp were sampled and dissected before the experiment and exposed for 2 h, 6 h, 1 d, 3 d, 5 d, 7 d, 14 d and 28 d, and the sampling process was the same as that for the short-term exposure test. Three parallel groups were set up, and each group included a control group and 2 exposed groups.

### 2.3. Sample Pretreatment

Goldfish tissue samples were placed into a 20 mL centrifuge tube and mixed with 12 mL ethyl acetate aqueous solution (*v*/*v* = 2/1). The mixture was vortexed for 1 min, and then 1 g anhydrous MgSO_4_ and 0.5 g diatomite were added, followed by another vortex and mixing for 1 min. The sample was sonicated at room temperature for 10 min and then centrifuged at 4000 r/min at 4 °C for 10 min. The upper layer supernatant was collected, dried using nitrogen gas, reconstituted with 1 mL of methanol, and subjected to membrane analysis.

### 2.4. Instrument Conditions

The HPLC column used was an Eclipse C_18_ column (1.8 μm, 3.0 × 100 mm, Agilent, Santa Clara, CA USA) with a column temperature of 40 °C, injection volume of 2 µL, and flow rate of 5 μL/s. The mobile phase consisted of 0.1% formic acid water (aqueous phase) and methanol (organic phase) using gradient elution. The elution procedure was as follows (Table 1).

The mass spectrometry was performed using an electrospray ion source (ESI) in the positive ion mode, and the select ion reaction monitoring mode (SRM) was used for scanning. The spray voltage was set at 4000 V, and the temperature was maintained at 200 °C. High-purity nitrogen was used as both the sheath gas and auxiliary gas at a pressure of 60 psi, while the ion transport capillary temperature was set at 450 °C. The collected fragments are summarized below (Table 2).

### 2.5. Detection Limit, Precision and Recovery

The method used in this study was evaluated for its detection limit, limit of quantification, precision, and recovery, and the results showed that it met the detection requirements with good reproducibility and high precision (Table 3).

### 2.6. Data Analysis

Microsoft Excel software (V2019, Microsoft, Redmond, WA, USA) was used to process the data, calculate the mean value and standard deviation of the data, and conduct *t*-test analyses. (*p* < 0.05 indicated a significant difference). The results of each group are displayed by means of ±standard error (SEM) and error bars.

## 3. Results

### 3.1. Concentration Changes of IMI and Its Metabolites in Goldfish under Short-Term Exposure

**Figure 1:**
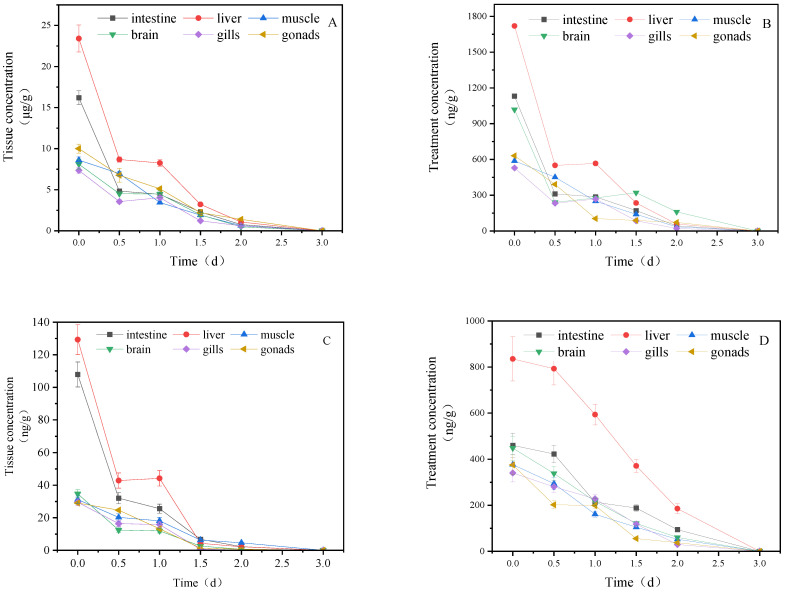
Concentration changes of IMI and its metabolites in goldfish under short-term exposure (treatment concentration: 40 mg/L): (**A**) IMI; (**B**) IMI-urea; (**C**) 6-chloronicotinic acid; (**D**): 5-hydroxy-IMI.

Figure 1 illustrates the concentration changes of IMI and its metabolites in goldfish tissues under short-term exposure. IMI accumulated in goldfish after one day of exposure, with the highest concentration in the liver (23.411 μg/g_tissue_), followed by the intestine (16.20 μg/g_tissue_), muscle (8.61 μg/g_tissue_), gill (7.36 μg/g_tissue_), gonads (7.41 μg/g_tissue_) and brain (7.15 μg/g_tissue_). After transfer to clean water, the concentration of IMI and its metabolites in all tissues showed a decreasing trend, and after 3 days, the content in all tissues was lower than the detection limit.

### 3.2. Concentration Changes of IMI in Goldfish under Continuous Exposure

**Figure 2:**
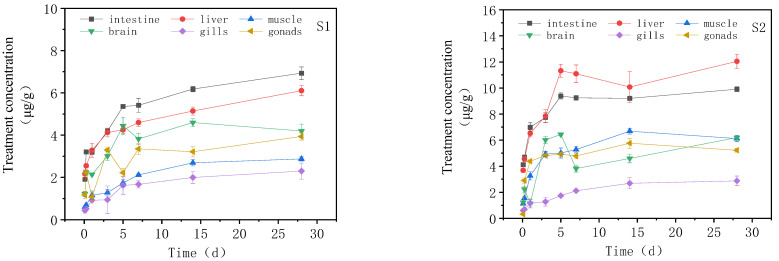
Concentration changes of IMI in goldfish under continuous exposure (treatment concentration: (**S1**) 20 mg/L; (**S2**) 40 mg/L).

The changes in the IMI concentration in different tissues of goldfish with exposure time are presented in Figure 2 during the continuous exposure treatment test. There were significant differences in the distribution of IMI in the muscle, liver, intestine, brain tissue, muscle and gonad (*p* < 0.05). In the high concentration treatment group, the IMI accumulation increased rapidly in each tissue at the beginning of the treatment, reaching a peak on day 5. Subsequently, from day 5 to day 28, the accumulation rate of IMI in each tissue declined and eventually reached a steady state. The intestinal tract and muscle even showed a downward trend between days 5 and 14. Finally, after 28 days of exposure, the accumulation of IMI in goldfish tissues from high to low was the liver (12.040 μg/g_tissue_), intestine (9.91 μg/g_tissue_), muscle (6.20 μg/g_tissue_), gill (6.11 μg/g_tissue_), gonads (5.22 μg/g_tissue_) and brain (2.87 μg/g_tissue_). In the low concentration treatment group, the accumulation of IMI was similar to that in the high concentration treatment group. In the low concentration treatment group, the accumulation of IMI was similar to that in the high concentration treatment group, with rapid accumulation in the tissue at 0–5 d, and then the accumulation at 5–28 d was still in an upward trend, but the rising speed gradually decreased. The amount of IMI accumulated in various tissues from high to low was the intestine (6.93 μg/g_tissue_), liver (6.10 μg/g_tissue_), muscle (4.20 μg/g_tissue_), gonads (3.93 μg/g_tissue_), gill (2.81 μg/g_tissue_) and brain (2.30 μg/g_tissue_).

### 3.3. Dynamic Changes in IMI Metabolites in Different Tissues of Goldfish under Continuous Exposure

#### 3.3.1. Concentration Changes in IMI Metabolites in Gill Tissues

**Figure 3:**
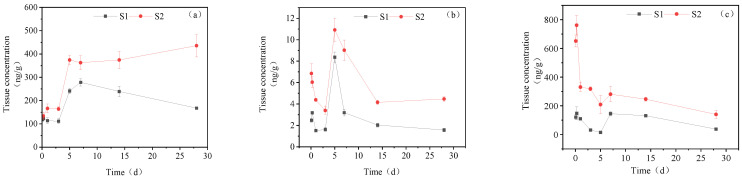
Concentration changes in IMI metabolites in gill tissues (treatment concentration: (S1) 20 mg/L; (S2) 40 mg/L): (**a**) IMI-urea; (**b**) 6-chloronicotinic acid; (**c**) 5-hydroxy-IMI.

Figure 3 illustrates the presence of IMI-urea, 6-chloronicotinic acid and 5-hydroxy-IMI in the gill tissue of goldfish following exposure to IMI. In the high concentration treatment group, the concentration of IMI-urea increased in the gill tissue, ultimately reaching 435.59 ng/g_tissue_ at 28 days. The accumulation of IMI-urea was relatively gradual over the first 3 days, with a rapid increase to 374.30 ng/g_tissue_ at day 5, followed by a slow increase to its maximum concentration between days 5 and 28. The concentration of 6-chloronicotinic acid initially decreased, subsequently increased, and then decreased again in the gill tissue. At days 0–3, its concentration declined from 6.85 ng/g_tissue_ to 3.40 ng/g_tissue_, followed by an increase to a maximum of 10.92 ng/g_tissue_ at day 5, which then decreased to 4.47 ng/g_tissue_ by day 28. The maximum accumulation of 5-hydroxy-IMI was observed at 761.24 ng/g_tissue_ at 6 h, followed by a downward trend until day 28, with a final concentration of 140.48 ng/g_tissue_.

In the low concentration treatment group, the accumulation of IMI-urea exhibited a similar trend as observed in the high concentration group, with a slight increase from 0 to 3 d, followed by a decline after peaking at 278.26 ng/g_tissue_ in 3–7 d. The accumulation of IMI-urea was 167.51 ng/g_tissue_ at 28 d. In the 0–28 d period, the content of 6-chloronicotinic acid exhibited two peaks at 6 h and 5 d post-treatment, with concentrations of 3.18 ng/g_tissue_ and 8.37 ng/g_tissue_, respectively. The final accumulation was 1.57 ng/g_tissue_ at 28 d. The maximum accumulation of 5-hydroxy-IMI reached 148.22 ng/g_tissue_ at 6 h and showed a downward trend from 6 h to 5 d. Once it decreased to a certain concentration, the content of 5-hydroxy-IMI suddenly increased on the 7th day and then began to decline. Ultimately, the concentration of 5-hydroxy-IMI was 37.69 ng/g_tissue_ on the 28th day.

#### 3.3.2. Dynamic Changes in IMI Metabolites in the Intestine

**Figure 4:**
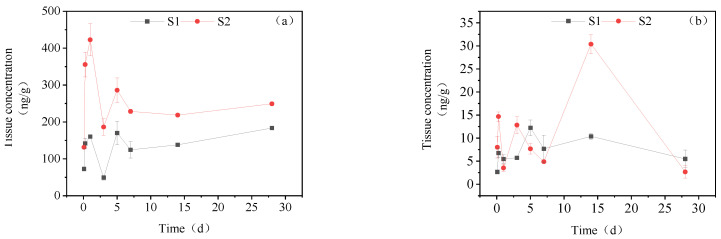

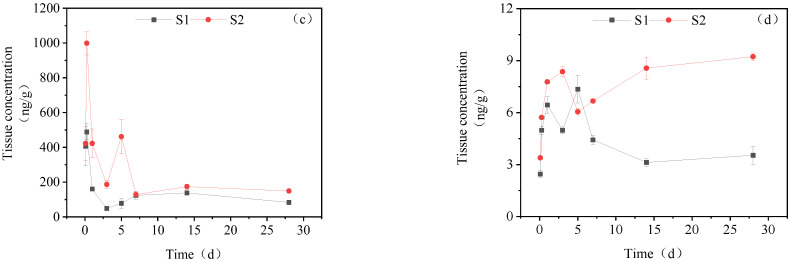
Concentration changes in IMI metabolites in the intestine (treatment concentration: (S1) 20 mg/L; (S2) 40 mg/L): (**a**) IMI-urea; (**b**) 6-chloronicotinic acid; (**c**) 5-hydroxy-IMI; (**d**) IMI-olefin.

Figure 4 illustrates that, in addition to IMI-urea, 5-hydroxy-IMI and 6-chloronicotinic acid, IMI was detected in the intestine after IMI exposure. The maximum accumulation of these metabolites, from high to low, was observed for 5-hydroxy-IMI, IMI-urea, 6-chloronicotinic acid and IMI-olefin. In the high concentration treatment group, IMI-urea showed an increasing trend until 1 day, reaching a maximum value of 422.77 ng/g_tissue_, followed by a gradual decline, and it finally stabilized at a concentration of 249.23 ng/g_tissue_. The dynamics of accumulation of 6-chloronicotinic acid were more complex, with an initial increase and subsequent decrease observed in 2 h–1 d, 1–7 d and 7–28 d. The maximum accumulation was observed at 14 d (30.37 ng/g_tissue_), while the final concentration was 2.67 ng/g_tissue_ at 28 d. The content of 5-hydroxy-IMI peaked at a value of 999.02 ng/g_tissue_ at 6 h, followed by a declining trend, reaching a certain level at 3 d, then increasing to 461.78 ng/g_tissue_ at 3–5 d, and finally stabilizing at a concentration of 149.23 ng/g_tissue_ at 28 d. The content of IMI-olefin increased until 3 days, then decreased to 6.05 ng/g_tissue_ on the 5th day, and then gradually increased again. Finally, the content of IMI-olefin was 9.23 ng/g_tissue_ at 28 d.

In the low concentration treatment group, the accumulation of IMI-urea was comparable to that in the high concentration treatment group, with an accumulation of 183.54 ng/g_tissue_ observed at 28 days. The concentration of 6-chloronicotinic acid demonstrated an overall increase within the first 5 days, with a maximum of 12.24 ng/g_tissue_ on day 5, followed by a decline. The final concentration of 6-chloronicotinic acid was 5.49 ng/g_tissue_ at 28 days, which was higher than that observed in the high concentration treatment group. The concentration of 5-hydroxy-IMI was similar to that in the high concentration treatment group, with a maximum accumulation of 488.64 ng/g_tissue_ and a final concentration of 83.54 ng/g_tissue_ at 28 days. The accumulation dynamics of IMI were similar to those of 6-chloronicotinic acid, with a maximum accumulation of 3.53 ng/g_tissue_.

#### 3.3.3. Dynamic Changes in IMI Metabolites in the Liver

**Figure 5:**
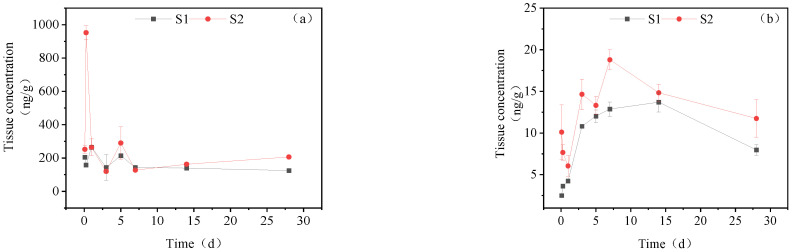

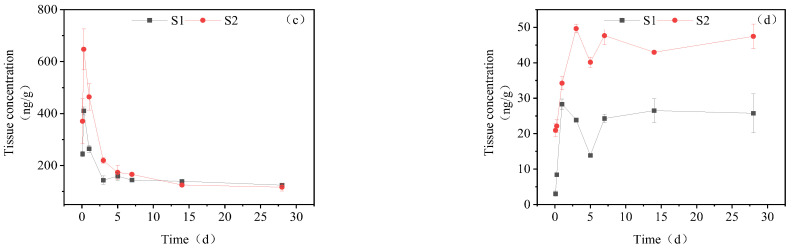
Concentration changes in IMI metabolites in intestine (treatment concentration: (S1) 20 mg/L; (S2) 40 mg/L): (**a**) IMI-urea; (**b**) 6-chloronicotinic acid; (**c**) 5-hydroxy-IMI; (**d**) IMI-olefin.

The results depicted in Figure 5 indicate that the metabolites of IMI in the liver are consistent with those in the intestine. In the high concentration treatment group, the maximum content of each metabolite was in the following order: IMI-urea, 5-hydroxy-IMI, IMI-olefin and 6-chloronicotinic acid. The accumulation dynamics of IMI-urea and 5-hydroxy-IMI were similar, with their concentrations reaching a maximum at 6 h after exposure, 952.38 ng/g_tissue_ and 653.13 ng/g_tissue_, respectively, and then reaching a relative equilibrium state from 6 h to 28 d. Finally, the concentration distribution was 206.33 ng/g_tissue_ and 156.27 ng/g_tissue_ at 28 d. The content of 6-chloronicotinic acid showed a zigzag upward trend within 0–7 d, reached a maximum accumulation of 18.79 ng/g_tissue_ at 7 d, and began to decline. The final concentration was 12.24 ng/g_tissue_ at 28 d, which was lower than that of the low concentration treatment group. The concentration of IMI increased from 0 to 3 days, reaching a maximum of 49.65 ng/g_tissue_ at 3 days and remaining relatively stable after the concentration decreased at 5 days.

In the low concentration treatment group, the metabolites of IMI in the liver were found to follow the same pattern as that of the high concentration treatment group. The maximum accumulation amounts of each metabolite in the low concentration group were 5-hydroxy-IMI, IMI-urea, IMI-olefin, and 6-chloronicotinic acid, in descending order. The concentration changes of IMI-urea and 5-hydroxy-IMI were similar to those in the high concentration group, with maximum accumulation amounts of 271.29 ng/g_tissue_ and 423 ng/g_tissue_, respectively, at 6 h. The content of 6-chloronicotinic acid showed an increasing trend before 14 days, reaching a maximum of 13.76 ng/g_tissue_ at 14 days and then decreasing at 28 days. The concentration of IMI showed an upward trend in the first day, accumulated to the maximum value of 28.32 ng/g_tissue_, and began to decrease in the following 1–5 days. The concentration then stabilized at 24.32 ng/g_tissue_ from 7–28 days.

#### 3.3.4. Dynamic Changes in IMI Metabolites in Muscle

**Figure 6:**
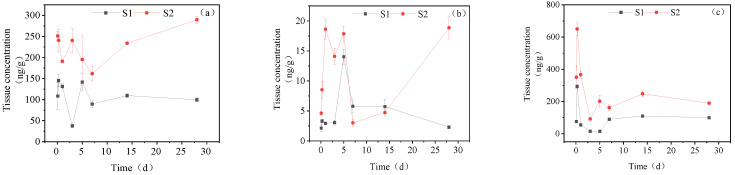
Concentration changes in IMI metabolites in muscle (treatment concentration: (S1) 20 mg/L; (S2) 40 mg/L): (**a**) IMI-urea; (**b**) 6-chloronicotinic acid; (**c**) 5-hydroxy-IMI.

Figure 6 illustrates the accumulation dynamics of three metabolites of IMI in goldfish brain tissue. The metabolites’ maximum content in the brain tissue was 5-hydroxy-IMI, IMI-urea, and 6-chloronicotinic acid, in descending order. In the high concentration treatment group, the concentration of IMI-urea in goldfish muscle tissue fluctuated, with a maximum accumulation of 289.50 ng/g_tissue_ at 28 d. The concentration of 6-chloronicotinic acid in muscle tissue ranged between 3.06 and 18.86 ng/g_tissue_. The concentration of 5-hydroxy-IMI reached its maximum of 650.35.45 ng/g_tissue_ at 6 h, then decreased to a certain level and remained relatively stable, with a final concentration of 189.50 ng/g_tissue_ at 28 d. In the low concentration treatment group, the accumulation dynamics of IMI-urea and 5-hydroxy-IMI were similar to those in the high concentration treatment group. However, in the low concentration treatment group, the concentration of 6-chloronicotinic acid first increased and then decreased, reaching a maximum accumulation of 14.03 ng/g_tissue_.

#### 3.3.5. Dynamic Changes in IMI Metabolites in Brain Tissue

**Figure 7:**
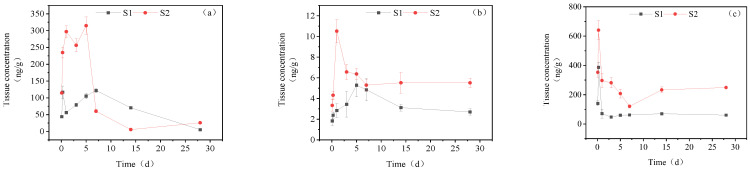
Concentration changes in IMI metabolites in brain tissue (treatment concentration: (S1) 20 mg/L; (S2) 40 mg/L): (**a**) IMI-urea; (**b**) 6-chloronicotinic acid; (**c**) 5-hydroxy-IMI.

Figure 7 presents the accumulation dynamics of IMI metabolites in goldfish brain tissue. Three metabolites of IMI were detected in the brain tissue, with 5-hydroxy-IMI, IMI-urea, and 6-chloronicotinic acid being the metabolites with the highest accumulation. In the high concentration treatment group, IMI-urea showed an increasing trend within 0–5 d, reaching a maximum accumulation of 314.76 ng/g_tissue_ at 5 d, and then decreased to a lower level at 14 d and 28 d. The concentration of 6-chloronicotinic acid peaked at 10.51 ng/g_tissue_ on the first day and then decreased to a stable level. The maximum accumulation of 5-hydroxy-IMI was 640.83 ng/g_tissue_ at 6 h, and its concentration then decreased with a final concentration of 249.21 ng/g_tissue_ at 28 d. In the low concentration treatment group, the three metabolites showed a trend of increasing first and then decreasing. The maximum content of IMI-urea was 121.91 ng/g_tissue_ on the 7th day, after which its concentration decreased. 6-Chloronicotinic acid reached a maximum of 5.28 ng/g_tissue_ on the 5th day, and the maximum accumulation of 5-hydroxy-IMI was 386.76 ng/g_tissue_ at 6 h.

#### 3.3.6. Dynamic Changes in IMI Metabolites in Gonads

**Figure 8:**
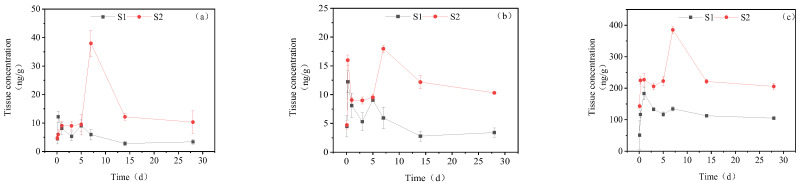
Concentration changes in IMI metabolites in gonads (treatment concentration: (S1) 20 mg/L; (S2) 40 mg/L): (**a**) IMI-urea; (**b**) 6-chloronicotinic acid; (**c**) 5-hydroxy-IMI.

Figure 8 presents the accumulation dynamics of IMI metabolites in Gonads. In the gonads of the goldfish, the concentration of IMI-urea was significantly lower than that of the other tissues, with its maximum content amounting to only one-tenth of that observed in the other tissues. In the high concentration treatment group, IMI-urea exhibited a steady rise before 0–5 d, followed by a sharp increase to a maximum value of 37.97 ng/g_tissue_ on the 7th day and, subsequently, returned to normal levels between the 14th and 28th days. 6-Chloronicotinic acid showed two peaks of 15.98 ng/g_tissue_ and 17.98 ng/g_tissue_ at 6 h and 7 d, respectively. The concentration of 5-hydroxy-IMI increased initially and then decreased, reaching a maximum accumulation of 385.21 ng/g_tissue_ at 7 days. In the low concentration treatment group, the concentrations of IMI-urea, 6-chloronicotinic acid and 5-hydroxy-IMI reached their maximum levels at 6 h, with concentrations of 12.22 ng/g_tissue_, 12.22 ng/g_tissue_ and 183.07 ng/g_tissue_, respectively. Subsequently, the concentrations of these three metabolites decreased from 6 h to 28 d.

## 4. Discussion

With the rapid growth of IMI applications, its own advantages have attracted wide attention. Previous studies have shown that IMI can cause various negative effects on nontarget aquatic organisms. However, in past studies, researchers often only paid attention to the toxicity of IMI in itself, and the metabolite toxicity of IMI is still less studied. Studies have shown that the toxicity of IMI to organisms is closely related to its metabolism. After the detoxification of IMI, the bioactivity of its metabolites is reduced, but in some cases, more active metabolites may be produced. When studying the toxic effects of IMI, not only IMI itself but also the toxicity of its metabolites should be considered. Therefore, this study preliminarily investigated the accumulation of IMI and its metabolites in goldfish through short-term and long-term exposure experiments.

In the short-term exposure experiment, it was observed that the content of IMI and its metabolites in all tissues was below the detection limit three days after transfer, which is consistent with the degradation rate of IMI in rats. After oral administration of IMI to rats, it was found that the initial half-life of IMI was approximately 3 h, while the final half-life ranged from 26 to 118 h, and the residual amount of IMI in tissues was less than 1% after 48 h [18]. Similarly, Poliserpi et al. reported that the concentration of IMI in the tissues of grayish baywing (*Agelaioides baduis*) was below the detection limit after 48 h of oral administration of IMI in the United States [19]. These findings suggest that IMI undergoes rapid degradation in organisms. The accumulation of chemicals in organisms is often dependent on their solubility, where better water solubility leads to poorer accumulation ability [20]. Because of its better water solubility, IMI and its metabolites have a faster degradation rate in goldfish. In contrast, the concentration of IMI in gills increased at 1–1.5 days. The gill is the primary sensing organ of fish for pollutants in water, and because of the lack of metabolic enzymes around it, the concentration of IMI in gill tissue should increase with an increasing exposure time [21]. Subsequently, water samples were collected at 1–1.5 days, and a certain amount of IMI was detected in the water samples at 1.5 days (unpublished data). This could be due to the IMI absorbed by goldfish being excreted through the intestine and then reabsorbed by the gills, resulting in a transient rise in the concentration in IMI in the gills.

The continuous exposure test revealed that goldfish accumulated the highest concentration of IMI in their intestine and liver. This result is consistent with previous studies, such as Yi Yang et al.’s research (2022), which found that after thiamethoxam exposure, zebrafish had the highest concentration of thiamethoxam in their liver and intestine, suggesting that the hepatointestinal system is a primary site of accumulation for exogenous drugs [22]. Similarly, Yang et al.’s study (2006) reported that the highest accumulation of IMI in crucian carp was found in the liver [23]. Therefore, it can be inferred that the accumulation pathway through hepatointestinal recycling may play an important role in IMI absorption in fish. Furthermore, the accumulation dynamics of IMI in the intestine, liver, gill tissue and brain tissue were relatively straightforward, with their content increasing with the duration of exposure. In contrast, the accumulation in muscle and gonad was more complex. Previous research has indicated that IMI accumulates in the muscle tissue of Procambarus clarkii, likely due to the high lipid content of the muscle composition and IMI’s lipophilicity. The presence of metabolic enzymes in muscle tissue leads to the degradation of IMI into low-toxicity metabolites, which enter the bloodstream and are excreted from the body [21]. The gonad is located on the dorsal wall of the body cavity of goldfish, near the end of the intestine. The accumulation of IMI in the gonad may be affected by multiple factors, including the intestine and blood, resulting in complex accumulation dynamics.

**Figure 9:**
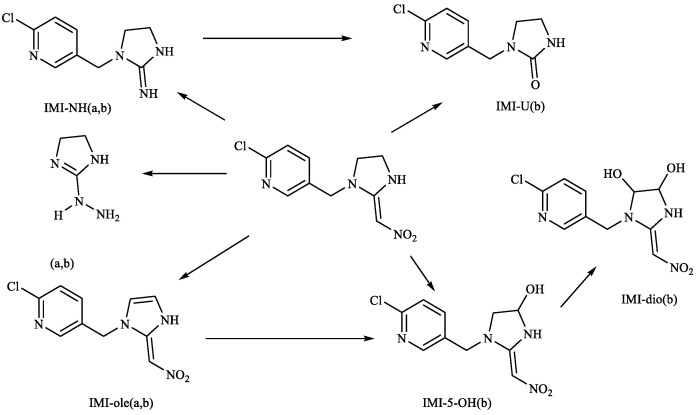
Metabolic pathways of IMI: (a) housefly: (b) honeybee.

Subsequently, we analyzed the timing of the maximum accumulation of IMI metabolites in each tissue and the ratio between the maximum accumulation and absorbed IMI. The results are presented in Table 4. It is evident that 5-hydroxy-IMI had the highest concentration in all tissues, followed by IMI-urea. Only a small amount of IMI was detected as 6-chloronicotinic acid and IMI-olefin, with the latter only present in the liver and intestine. This differs from Suchail et al.’s research on the distribution and metabolism of IMI in bees, where IMI-urea and 6-chloronicotinic acid were the main metabolites, especially in the midgut and rectum. IMI-olefin and 4,5-dihydroxy-IMI were preferentially produced in the head, chest and abdomen, which are rich in acetylcholine receptors [24]. The differences in this experiment’s distribution may be attributed to the concentration of IMI used in the treatment and the differences in metabolic pathways between vertebrates and invertebrates. Byren and Nishiwaki’s research showed that the primary metabolites of IMI after metabolism in houseflies and bees were 5-hydroxy-IMI, 4,5-dihydroxyIMI, 6-chloronicotinic acid, IMI-olefin and IMI-urea. See Figure 9 for the metabolic pathways [25,26].

**Figure 10:**
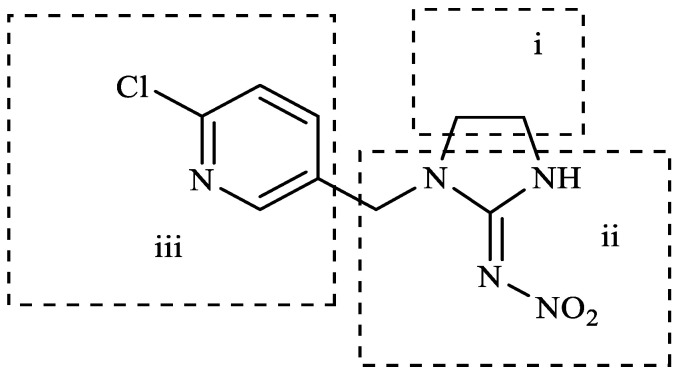
Structure of IMI.

**Figure 11:**
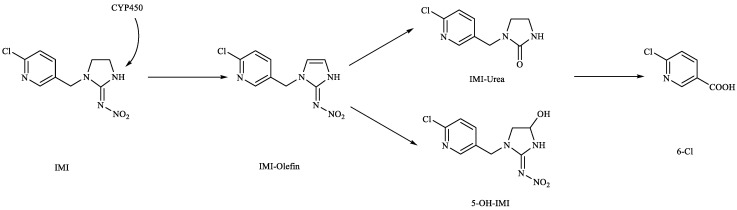
Possible metabolic pathways of IMI in goldfish.

In this study, the highest ratio of the total content of metabolites detected in the liver to IMI was observed at 6 h after exposure, indicating that the liver is likely the earliest metabolic site for IMI in goldfish. The intestine was also found to be an important site for IMI metabolism. These findings are consistent with a previous study by Yang et al., which showed that the metabolism of thiamethoxam in zebrafish occurred in both the liver and intestine and that its metabolic pathway involved N-demethylation and nitro reduction [23]. After exogenous drugs enter the organism, phase I and phase II metabolism occur under the action of catalytic enzymes in the body. Phase I metabolism mainly involves hydroxylation, desaturation, dealkylation and nitro reduction, among other reactions. The cytochrome CYP450 enzyme, which exists in the liver, is an important oxidative metabolic enzyme that catalyzes these reactions [27]. The structure of IMI, shown in Figure 10, contains chemical reaction sites located on the methylene bridge chain (i structure), the pharmacophore nitroimine (ii structure), and the six-membered ring (iii structure), which can undergo a series of metabolic reactions under the action of catalytic enzymes in vivo. Based on these observations, we speculate that the possible metabolic pathway of imidacloprid in goldfish is shown in Figure 11. After IMI is absorbed into the liver through the intestine, and under the action of the cytochrome CYP450 enzyme in the liver, it first dehydrogenates to produce IMI-olefin in structure (i). Because of the unstable chemical properties of IMI-olefin, metabolic reactions such as hydroxylation and nitro reduction, respectively, produce 5-hydroxy-IMI, and IMI-urea and 6-chloronicotinic acid. These metabolites enter tissues through the circulatory system for enrichment or exclusion.

Upon detoxification and metabolism, the biological activity of IMI metabolites will decrease, but in some cases, IMI may produce more active metabolites. For instance, Suchail’s study revealed that the two secondary metabolites of IMI in bees, IMI-olefin and 5-hydroxy-IMI, may have a greater relationship with the toxicity of IMI. The toxicity of IMI-olefin is twice that of IMI and 10 times that of 5-hydroxy-IMI [28]. Additionally, studies have reported that the toxicity of IMI-olefin to Bemisia tabaci and Myzus persicae is approximately 10 times and 16 times higher than that of IMI, respectively, indicating that IMI alkenyl has higher toxicity than the parent compound [29]. In this study, a small amount of IMI-olefin was detected in the intestine and liver of goldfish, and further exploration of its subsequent effects on the intestine and liver can help clarify the toxicity mechanism of IMI-olefin. Moreover, metabolites with nitroimine pharmacophores, such as hydroxylated IMI and IMI-olefin, are toxic to bees, while IMI urea and 6-chloronicotinic acid, which are metabolites without pharmacodynamic groups, are nontoxic to bees [30]. The biological activities of IMI and its metabolites, from high to low, are olefinic IMI, IMI-urea, 4-hydroxy-IMI, 5-hydroxy-IMI and 4,5-dihydroxy-IMI. Thus, the biological activity of IMI is produced by the interaction of the parent and metabolites. In-depth studies on the differences in the metabolic pathways of IMI in target and nontarget organisms, as well as its metabolic differences in different parts of the same organism, can provide a better understanding of its toxic mechanism and provide ideas and references for the standardized use of IMI.

## 5. Conclusions

In this study, HPLC–MS was used to investigate the tissue distribution of IMI and its metabolites in goldfish after short-term and continuous exposure. The results of the short-term exposure experiments indicated that after transferring the exposed goldfish to an IMI-free aqueous solution, the concentrations of IMI and its metabolites in various tissues decreased and were below the detection limit after 3 days. The concentrations of IMI and its metabolites varied among different treatment concentrations in the continuous exposure experiments. Among the IMI metabolites, 5-hydroxy-IMI and IMI-urea accumulated in equivalent amounts in various tissues, followed by 6-chloronicotinic acid. IMI-olefin was detected only in the intestine and liver. These results allow us to propose a possible metabolic pathway of IMI in goldfish. This study contributes to our understanding of the metabolic behavior of IMI in organisms and provides new data to support the investigation of its toxicity.

## Figures and Tables

**Table 1:** Gradient elution procedure.

Time/min	Aqueous Phase/%	Organic Phase/%
0–5	80	20
5	60	40
6	40	60
7	80	20
8	80	20

**Table 2:** Fragment parameters were collected using mass spectrometry.

Detection Object	Structural Formula	Parent Ion	Daughter Ion
IMI	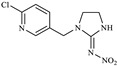	256.2	175.3
IMI-urea	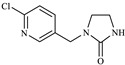	212.2	128.1
IMI-olefin	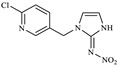	254.2	171.0
5-Hydroxy-IMI	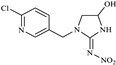	272.2	225.3
6-Chloronicotinic acid	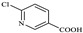	155.9	112.1

**Table 3:** Detection limit, precision and recovery of IMI and its metabolites.

Object	LOD (Limit of Detection) (μg/L)	LOQ (Limit of Quantitation) (μg/L)	Precision/%	Recovery/%	RSD/%
IMI	0.001	0.00334	3.41	96.08	6.523
IMI-urea	0.0099	0.032907	1.98	99.77	4.172
IMI-olefin	0.0011	0.003519	3.79	85.25	7.355
5-Hydroxy-IMI	0.0068	0.022718	2.84	87.24	8.825
6-Chloronicotinic acid	0.0056	0.018682	2.64	100.23	2.805

**Table 4:** The metabolites of IMI.

	Metabolite	Maximum Accumulation	Time	Ratio
Intestine	IMI-urea	432.34	24 h	7.47%
	IMI-olefin	9.37	28 d	0.10%
	6-Chloronicotinic acid	31.27	14 d	0.32%
	5-Hydroxy-IMI	1021.25	6 h	35.64%
Liver	IMI-urea	952.38	6 h	25.81%
	IMI-olefin	38.28	28 d	0.32%
	6-Chloronicotinic acid	18.79	14 d	0.18%
	5-Hydroxy-IMI	653.13	6 h	23.60%
Gill	IMI-urea	430.67	28 d	9.72%
	6-Chloronicotinic acid	10.97	5 d	0.23%
	5-Hydroxy-IMI	784.34	6 h	42.68%
Muscle	IMI-urea	298.39	28 d	3.99%
	6-Chloronicotinic acid	18.85	1 d	0.16%
	5-Hydroxy-IMI	758.45	6 h	19.34%
Brain	IMI–urea	319.24	5 d	7.30%
	6-Chloronicotinic acid	10.74	1 d	0.06%
	5-Hydroxy-IMI	654	6 h	21.80%
Gonad	IMI-urea	38.92	7 d	0.41%
	6-Chloronicotinic acid	18.34	6 h	0.59%
	5-Hydroxy-IMI	389.56	7 d	5.58%

## Data Availability

The data presented in this study are available upon request from the corresponding author.
